# Plasma fibrinogen may predict persistent infection before reimplantation in two-stage exchange arthroplasty for periprosthetic hip infection

**DOI:** 10.1186/s13018-019-1179-9

**Published:** 2019-05-14

**Authors:** Chi Xu, Peng-Fei Qu, Wei Chai, Rui Li, Ji-Ying Chen

**Affiliations:** Department of Orthopaedic Surgery, General Hospital of People’s Liberation Army, No. 28 Fuxing Road, Haidian District, Beijing, 100853 China

**Keywords:** Periprosthetic joint infection, Two-stage exchange arthroplasty, D-dimer, Fibrinogen

## Abstract

**Background:**

The diagnosis of persistent infection before reimplantation in two-stage exchange arthroplasty for periprosthetic joint infection (PJI) remains challenging. Currently, several studies suggested coagulation-related markers, such as D-dimer and fibrinogen, may be promising in diagnose of PJI. The purpose of the study was to investigate the predictive values of plasma D-dimer and fibrinogen for assessment of persistent infection before reimplantation hip arthroplasty.

**Methods:**

We retrospectively reviewed 129 hips that treated with two-stage exchange arthroplasty for PJI from 2012 to 2016 in our institution. The persistent infection before reimplantation was based on a modified Musculoskeletal Infection Society (MSIS) criteria. After exclusion, 102 hips were included in the final analysis. Receiver operating characteristic (ROC) curves were generated to determine the prognostic value of plasma D-dimer and fibrinogen in predicting persistent infection before reimplantation.

**Results:**

The area the under ROC curves (AUC) for fibrinogen (0.773; 95% confidential interval [CI], 0.569–0.905) was significantly higher than that of D-dimer (0.565; 95% CI, 0.329–0.777). With the calculated threshold of fibrinogen set at 3.61 g/L, the sensitivity, specificity, positive predictive value (PPV), and negative predictive value (NPV) was 87.5%, 62.8%, 16.7%, and 98.3%, respectively. With the threshold value of D-dimer set at 0.82 μg/mL, the sensitivity, specificity, PPV, and NPV was 83.3%, 41.9%, 21.7%, and 92.9%, respectively.

**Conclusions:**

In conclusion, the current study reveals that the plasma fibrinogen may be a promising biomarker in predicting persistent infection before reimplantation. Further prospective studies with larger cohorts are needed to validate predictive values and optimal thresholds of coagulation-related markers.

## Introduction

The management of periprosthetic joint infection (PJI) following total joint arthroplasty remains challenging. Although two-stage exchange arthroplasty has been the preferred treatment for chronic PJI in North America, the reported rate of re-infection is even up to 30% [1–3]. The rationale behind this unacceptable outcome could be the difficulty in exclusion of persistent infection before second-stage reimplantation [4–8].

Numerous attempts have been made to determine the eradication of infection before reimplantation. However, prior studies have suggested limited predictive values of the widely used biomarkers for diagnosis of PJI, including serum erythrocyte sedimentation rate (ESR) and C-reactive protein (CRP) [8, 9], synovial white blood cell (WBC) count and percentage of polymorphonuclear cells (PMN%) [7], aspiration culture [10], and histology analysis [11, 12]. Additionally, aspiration performed on hips with the antibiotic spacer can be hard with a high risk of “dry tap” despite the aid of fluoroscopic guidance [7, 13].

The literature has indicated the existence of closed correlations between the coagulation cascade and infection/inflammatory mechanisms [14, 15]. Recently, several studies have suggested the promising value of coagulation-related biomarkers, such as D-dimer and fibrinogen in the diagnosis of PJI [16, 17]. However, its ability to identify persistent infection at the time of reimplantation remains unknown.

The purpose of the present study was to investigate (1) the diagnostic values of plasma D-dimer and fibrinogen for assessment of persistent infection before reimplantation hip arthroplasty and (2) the best thresholds of D-dimer and fibrinogen that are predictive of persistent PJI.

## Methods

**Fig. 1:**
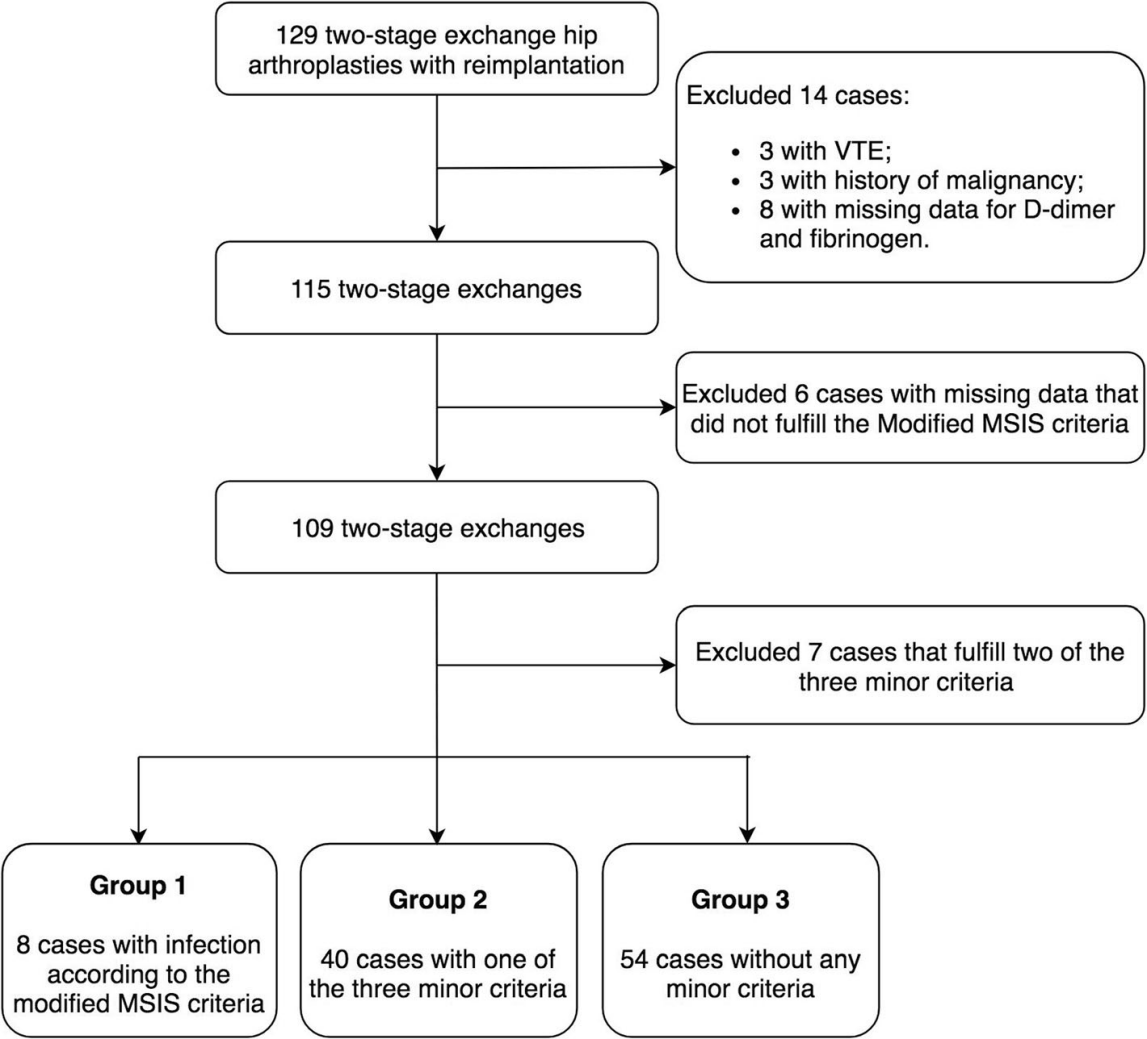
Flowchart

After the Institutional Review Board approval, we retrospectively reviewed all consecutive PJIs that were treated with two-stage exchange arthroplasty for periprosthetic hip infection from 2012 to 2016 in our institution. Of them, 129 two-stage exchanges (129 patients) were reimplanted without any surgeries between the first and second stage. The diagnosis of PJI was according to the Musculoskeletal Infection Society (MSIS) criteria [18]. Patients with a history of venous thrombus embolism (VTE), malignancy, and liver diseases and patients with missing critical data were excluded. As hip aspirations were not routinely performed prior to the second-stage reimplantation in our institution, the diagnosis of persistent infection was based on a modified MSIS criteria [7, 18]: the presence of a sinus tract communicating with the joint at surgery or two positive intraoperative periprosthetic cultures with the same organism or fulfill all the following three minor criteria including (1) ESR > 30 mm/h and CRP > 10 mg/L, (2) a single positive intraoperative periprosthetic tissue culture, and (3) a positive histologic analysis of periprosthetic tissue [> 5 neutrophils per high power field]. Seven cases that satisfied two of the minor criteria were considered as suspected infection and were also excluded. Finally, 8 hips (group 1) and 94 controls, including 40 hips with one of the minor criteria (group 2) and 54 cases without any minor criteria (group 3), were included in the final analysis (Fig. 1).

Plasma fibrinogen and D-dimer values were routinely obtained before reimplantation after admission. Fibrinogen and D-dimer levels were measured by STA-R Evolution® analyzer (Stago Diagnostica, Asnieres, France) and expressed in gram per liter (g/L) and microgram per milliliter (μg/mL), respectively. Medical records were reviewed manually to retrieve pertinent information including age, gender, body mass index (BMI), serum CRP and ESR values, intraoperative cultures, and histologic results.

An institutional standard protocol of two-stage exchange arthroplasty was performed. During the first-stage resection arthroplasty, all implanted components were removed followed by extensive debridement and irrigation. An articular antibiotic-loaded cement spacer, containing 6–10 g of vancomycin and 2–4 g of meropenem per 40 g bone cement (Heraeus Medical GmbH, Wehrheim/Ts., Germany), was then inserted. The combination of vancomycin and meropenem in the bone cement was utilized in accordance with our institutional infection control department, which explained that more than 90% of the organisms isolated from patients with PJI were sensitive to one or both antibiotics. Following resection arthroplasty, 4 weeks of prophylactic anticoagulation (Rivaroxaban, 5 mg PO q 24 h) and 6–8 weeks of systemic antibiotic therapy were prescribed. The selection of antibiotic application was based on culture sensitivity reports and institutional guidelines infectious specialists’ consultation. The timing of reimplantation was based on the following criteria: no clinical signs of infection, a well-healed surgical wound, and gradually decreased of ESR and CRP. Fibrinogen and D-dimer values were not used to make decisions. All patients had a period of at least 2-week antibiotic holiday before second-stage reimplantation. During the second-stage surgery, antibiotic-loaded cement spacer was removed. Four to six tissue samples for culture and three to five samples for histology analysis were taken from the periprosthetic membrane and other periprosthetic tissues in which infection was suspected. Then, the prostheses were implanted followed by re-debridement and irrigation. Parenteral antibiotics were given postoperatively until the intraoperative cultures were negative findings.

### Statistical analysis

All of the statistical analyses were performed with the statistical software packages R (http://www.R-project.org, The R Foundation). Categorical variables were presented as frequencies and percentages and continuous variables as means and standard deviation or median and range. The differences between groups were compared with the use of the Mann-Whitney test for continuous variables and the Fisher’s exact test for categorical variables. Patient characteristics were shown in Table 1. There was no significant difference in age, gender, and BMI between groups.

**Table 1:** Patient characteristics

	Group 1 (*n* = 8)	Group 2 (*n* = 40)	Group 3 (*n* = 54)	*p* value
Age (year)	53.3 ± 14.9	51.4 ± 17.5	56.5 ± 14.8	0.333
Gender				0.100
Male	5 (62.5%)	13 (32.5%)	28 (51.9%)	
Female	3 (37.5%)	27 (67.5%)	26 (48.1%)	
BMI (kg/m^2^)	24.6 ± 4.0	25.3 ± 3.5	23.5 ± 3.6	0.125

*BMI* body mass index

Receiver operating characteristic (ROC) curves were generated using bootstrap resampling (times = 500) [19] to determine the performance of plasma fibrinogen and D-dimer in predicting persistent infection at the time of reimplantation. The area under the ROC curve (AUC) with 95% CI was used as a measure of diagnostic accuracy. AUCs were compared by using the DeLong method [20]. A *p* value of 0.05 was considered significant.

### Sensitivity analysis

For no gold standard criteria for persistent infection at reimplantation, we performed a set of sensitivity analyses to further validate our results. We reevaluated the diagnostic value of plasma fibrinogen and D-dimer by only using group 1 as infection cases and group 3 (54 cases without any minor criteria) as controls to validate results. Forty hips with one of the minor criteria in group 2 were excluded in the sensitivity analysis. Then, the ROC curves were made and AUCs were compared according to the aforementioned methods.

## Results

**Fig. 2:**
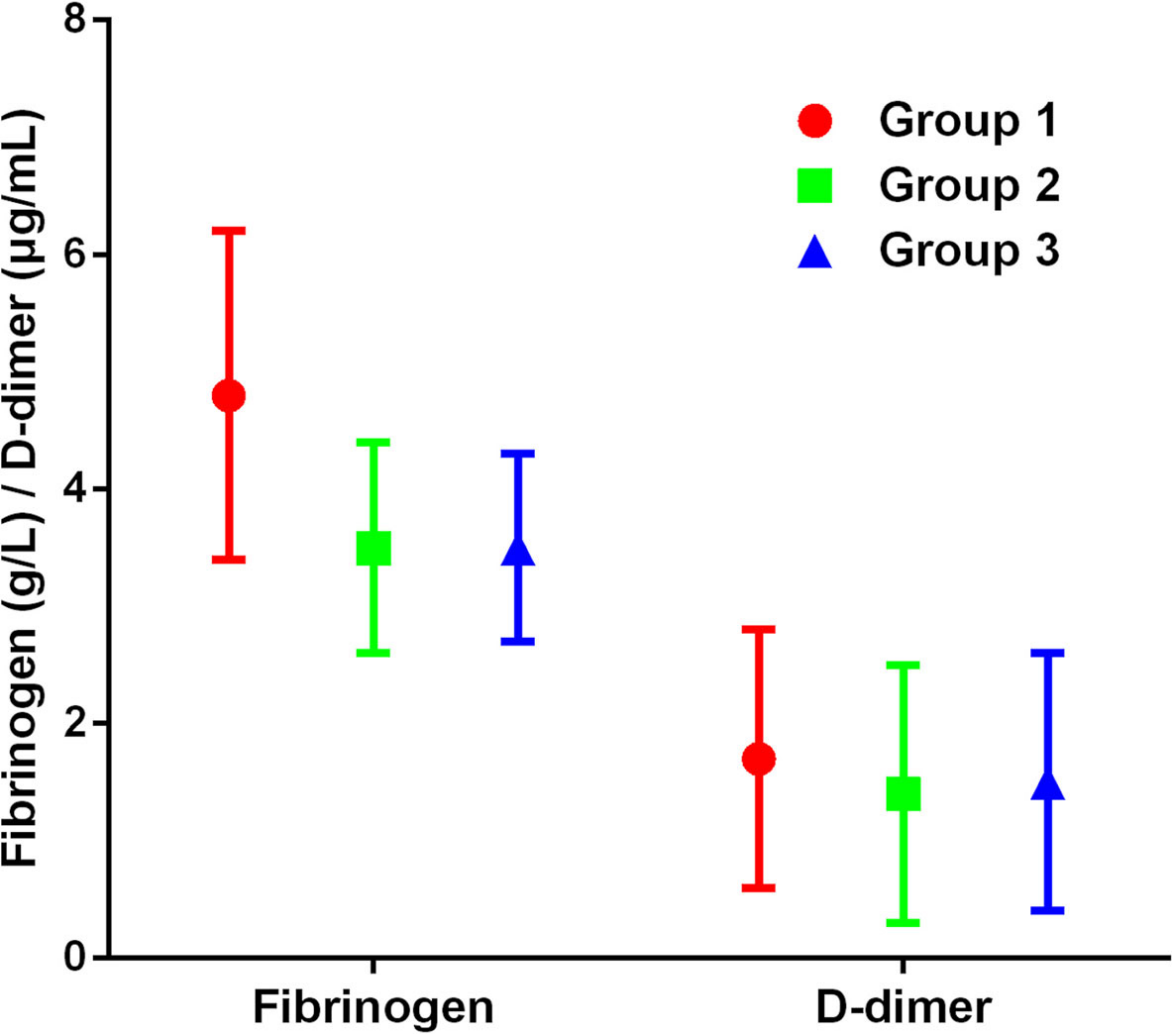
Plasma fibrinogen and D-dimer levels among groups

The plasma fibrinogen level was significantly higher in the patients with persistent infection (*p* = 0.032; Fig. 2); the median fibrinogen level was 4.3 g/L (range 3.0–6.9 g/L) in group 1 compared with 3.3 g/L (range 2.2–6.6 g/L) in group 2 and 3.4 g/L (range 2.1–6.7 g/L) in group 3. There was no difference in D-dimer level among groups (*p* = 0.745; Fig. 2); the median D-dimer level of groups 1, 2, and 3 was 1.6 μg/mL (range 0.3–3.2 μg/mL), 1.1 μg/mL (range 0.3–3.5 μg/mL), and 1.2 μg/mL (range 0.4–3.7 μg/mL), respectively.

**Fig. 3:**
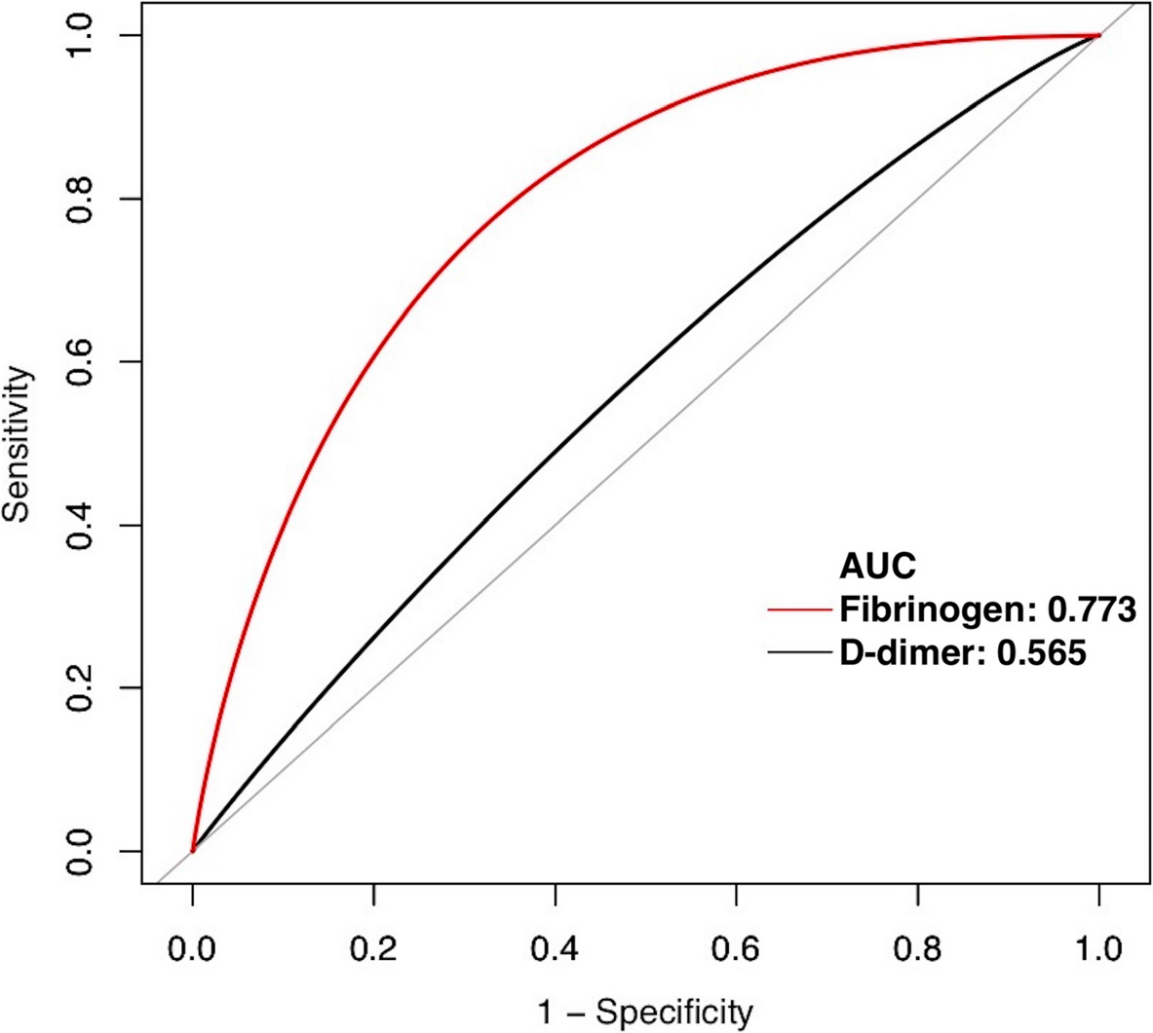
Receiver operating characteristic (ROC) curves for fibrinogen and D-dimer in predicting persistent infection at the time of reimplantation

Using the modified MSIS criteria indicating the persistent infection at the second-stage reimplantation, the AUC for fibrinogen (0.773; 95% CI, 0.569–0.905) was significantly higher than that of D-dimer (0.565; 95% CI, 0.329–0.777) (Fig. 3). With the calculated threshold of fibrinogen set at 3.61 g/L, the sensitivity, specificity, positive predictive value (PPV), and negative predictive value (NPV) was 87.5%, 62.8%, 16.7%, and 98.3%, respectively. With the threshold value of D-dimer set at 0.82 μg/mL, the sensitivity, specificity, PPV, and NPV were 83.3%, 41.9%, 21.7%, and 92.9%, respectively (Table 2).

**Table 2:** Values of fibrinogen and D-dimer in predicting persistent infection before reimplantation

	AUC	95% CI	Threshold	Sensitivity	Specificity	Positive predictive value	Negative predictive value
Fibrinogen	0.773	0.569–0.905	3.61 g/L	87.5%	62.8%	16.7%	98.3%
D-dimer	0.565	0.329–0.777	0.82 μg/mL	83.3%	41.9%	21.7%	92.9%
Sensitivity analysis							
Fibrinogen	0.778	0.558–0.917	3.61 g/L	87.5%	63.0%	25.9%	97.1%
D-dimer	0.565	0.318–0.785	0.82 μg/mL	83.3%	43.8%	35.7%	87.5%

*AUC* area of under the receiver operating characteristic curve

### Sensitivity analysis

In the first set of sensitivity analysis, only hips (54 cases in group 3) without any minor criteria (as per above) were considered as infection eradication at the time of reimplantation. The results of sensitivity analysis were consistent with above results; the AUC for fibrinogen (0.778; 95% CI, 0.558–0.917) was significantly higher than that of D-dimer (0.565; 95% CI, 0.318–0.785) (Table 2).

## Discussion

It is critical to identify persistent infection before second-stage reimplantation. However, the optimal timing of reimplantation remains unknown due to the lack of reliable biomarkers that can monitor persistent infection. The current study revealed that plasma fibrinogen levels might predict persistence of infection at the time of reimplantation with the best threshold of 3.61 g/L. Besides, D-dimer failed to indicate benefits in predicting infection eradication.

However, when interpreting our findings, several limitations should be considered. First, it was retrospective in nature and hence was subject to the inadequacy and variability in data collection. Second, it was a single-institution study with a limited sample size and, as a result, has limited external validity. Nevertheless, bootstrap resampling (*n* = 500), an internal validation method, was conducted in the present study to enlarge simple sizes statistically. Third, while the modified MSIS criteria were used in the current study, there has been no “gold standard” for diagnosis of persistent infection at the time of reimplantation. It is possible that cases with one minor criteria deemed to be not infected might have been infected. Nonetheless, a set of sensitivity analysis by using patients without any minor criteria as controls was performed to minimize bias. Although several studies defined persistence of infection by using treatment failure following reimplantation [5, 6, 21], it was not applied in this study as it might be not reasonable. For example, the persistence of infection before reimplantation can be eradicated during the second-stage procedure, or vulnerable patients without persistent infection at the time of reimplantation may suffer from re-infection postoperatively. Fourth, prophylactic anticoagulation following the first-stage resection arthroplasty may influence the coagulation biomarker values, such as D-dimer and fibrinogen. However, all the second-stage reimplantation was performed after more than 4 weeks of Rivaroxaban withdrawal, which had limited impact on the plasma D-dimer and fibrinogen levels. Lastly, the application of current results was limited in patients without VTE and malignancy. Lastly, it is difficult to present AUCs for ESR and CRP because they are part of the elements used in the reference standard diagnosis.

To our best knowledge, this is the first study to evaluate the predictive values of coagulation-related markers in the diagnosis of persistent infection at the time of reimplantation. Close connections between fibrinolytic activities and infection/inflammatory have been proved in the literature [14, 15, 22–24]. Fibrinogen is the precursor of fibrin that is a soluble glycoprotein weighing 340 kDa. It is composed of three polypeptide chains called Aα, Bβ, and γ and is synthesized in the liver [15]. While it has been well known for its crucial role in the coagulation cascade, fibrinogen also plays a key role in activating and mediating the inflammation process [14, 15]. A most recent study by Klim et al. has suggested fibrinogen may be promising in the diagnosis of PJI with a sensitivity of 0.90 and a specificity of 0.34 [16]. However, further studies are required to validate the value of fibrinogen in the diagnosis of PJI.

Additionally, Busso et al. have described the mechanisms that D-dimer levels were elevated in patients with rheumatoid arthritis [25]. Currently, a retrospective study indicated D-dimer outperformed both serum ESR and CRP in the diagnosis of PJI, with a sensitivity of 89% and a specificity of 93% [17]. Furthermore, they described five patients with raised D-dimer before reimplantation and two of them had a positive culture during reimplantation and then experienced failure due to infection postoperatively. However, serum ESR and CRP levels were normal in these two patients; hence, they speculated that D-dimer might be also valuable in predicting persistent infection before second-stage reimplantation. Unfortunately, the current study failed to identify the benefits of D-dimer in predicting persistent infection before reimplantation. The rationale may be a larger sample size used and different criteria for persistent infection in the present study.

Several studies have suggested classical markers used in the diagnosis of PJI, including serum ESR, CRP, synovial WBC count, and PMN%, performed limited values for assessment of persistent infection before reimplantation [7–9]. Newman et al. suggested that a high incidence of “dry tap” in patients with antibiotic cement spacers of the hip. Moreover, they indicated aspirations performed with saline lavage were not reliable in predicting persistent infection [7]. Currently, a single-institution study by Kheir et al. revealed that a positive leukocyte esterase (LE) strip test (2+) might be indicative of persistence of infection and resulted in a higher rate of subsequent failure [5]. The AUC of LE they reported was only 0.632, which was much lower than that of plasma fibrinogen (0.773) in the current study. However, this dissimilarity may be due to the different definitions for persistent infection and cohorts used.

## Conclusions

In conclusion, the current study reveals that the plasma fibrinogen may be a promising biomarker in predicting persistent infection before reimplantation. However, D-dimer may have limited benefit in predicting infection eradication. Further prospective studies with larger cohorts are required to validate predictive values and optimal thresholds of coagulation-related markers.

## Abbreviations

AUC: The area under the ROC curve
BMI: Body mass index
CRP: C-reactive protein
ESR: Erythrocyte sedimentation rate
LE: Leukocyte esterase
MSIS: Musculoskeletal Infection Society
NPV: Negative predictive value
PJI: Periprosthetic joint infection
PMN%: Percentage of polymorphonuclear cells
PPV: Positive predictive value
ROC: Receiver operating characteristic
VTE: Venous thrombus embolism
WBC: White blood cell
